# Cultured bloodstream *Trypanosoma brucei* adapt to life without mitochondrial translation release factor 1

**DOI:** 10.1038/s41598-018-23472-6

**Published:** 2018-03-23

**Authors:** Michaela Procházková, Brian Panicucci, Alena Zíková

**Affiliations:** 1 Institute of Parasitology, Biology Centre ASCR, Ceske Budejovice, Czech Republic; 2 Faculty of Science, University of South Bohemia, Ceske Budejovice, Czech Republic; 3 Present Address: Central European Institute of Technology, Masaryk University, Brno, Czech Republic

## Abstract

*Trypanosoma brucei* is an extracellular parasite that alternates between an insect vector (procyclic form) and the bloodstream of a mammalian host (bloodstream form). While it was previously reported that mitochondrial release factor 1 (TbMrf1) is essential in cultured procyclic form cells, we demonstrate here that *in vitro* bloodstream form cells can tolerate the elimination of TbMrf1. Therefore, we explored if this discrepancy is due to the unique bioenergetics of the parasite since procyclic form cells rely on oxidative phosphorylation; whereas bloodstream form cells utilize glycolysis for ATP production and F_o_F_1_-ATPase to maintain the essential mitochondrial membrane potential. The observed disruption of intact bloodstream form F_o_F_1_-ATPases serves as a proxy to indicate that the translation of its mitochondrially encoded subunit A6 is impaired without TbMrf1. While these null mutants have a decreased mitochondrial membrane potential, they have adapted by increasing their dependence on the electrogenic contributions of the ADP/ATP carrier to maintain the mitochondrial membrane potential above the minimum threshold required for *T. brucei* viability *in vitro*. However, this inefficient compensatory mechanism results in avirulent mutants in mice. Finally, the depletion of the codon-independent release factor TbPth4 in the TbMrf1 knockouts further exacerbates the characterized mitchondrial phenotypes.

## Introduction

Not only are *Trypanosoma brucei* medically and economically important parasites that cause disease in humans and livestock, but these flagellated protists are also an excellent model to address fundamental questions within eukaryotic cell biology^1^. One of the most prominent areas of research over the last three decades has focused on the mitochondrial (mt) gene expression of this early divergent eukaryote. Its mt DNA is arranged in an immense network of concatenated large (maxi-) and small (mini-) circular DNA molecules that are condensed into a disc-like structure termed the kinetoplast^2^. Transcription of the maxicircle DNA generates polycistronic precursors that are processed into two ribosomal RNAs and 18 mRNAs that encode subunits of the mitoribosome and the oxidative phosphorylation complexes. However, to generate functional open reading frames, 12 of these protein-encoding transcripts require further maturation by an RNA editing process that specifically directs the insertion or deletion of uridylate nucleotides (nt)^3^ with the help of short guide RNAs transcribed from the minicircles^4,5^. While much of the editing process has been elucidated, little is known about how the *T. brucei* mitoribosome selectively translates only correctly edited transcripts^6,7^.

Although mitoribosomes are more closely related to bacterial ribosomes than their eukaryotic cytosolic counterparts, they nonetheless possess shorter mt ribosomal RNA (rRNA) and have acquired additional mitochondrial-specific proteins. The *T. brucei* mitoribosome represents an extreme example of this evolutionary divergence as it consists of 133 subunits, 56 of which do not have any recognizable homology to components of known mitoribosomes outside the Kinetoplastida group^8^. Furthermore, the 611 nt 9S rRNA of the mitoribosomal small subunit (SSU) and the 1149 nt 12S rRNA of the large subunit (LSU) are extremely short^9,10^. The cryo-EM map of a kinetoplastid mitoribosome illustrates that proteins have replaced the loss of key functional rRNA helices^11^. While the overall size and morphology is comparable to the bacterial ribosome, the structure on average is more porous and the topology of the intersubunit space is remodeled. Furthermore, since the mRNA channel, the tRNA corridors and the nascent polypeptide exit tunnel are all predominantly lined with unique kinetoplastid proteins, it suggests that the specific translational mechanisms employed by the kinetoplastid mitoribosome will vary from the ribosomes examined in other model organisms^11^.

Noticeably, the *T. brucei* mt DNA does not encode for any tRNAs; instead, the full complement must be imported from nuclear encoded genes^12,13^. However, the mt genome does contain a cryptogene for a single ribosomal protein, a homolog of the highly conserved bacterial S12, designated RPS12^14^. The transcript is extensively edited throughout the open reading frame before it is translated. In the bacterial ribosome, S12 is incorporated into the shoulder of the SSU, near the subunit interface and extends into the decoding center of the SSU aminoacyl tRNA site (A site), where it binds 16S rRNA and plays a critical role in the fidelity of tRNA selection^15–17^. In addition, through its interaction with elongation factors, it acts as a control element in the translocation of the mRNA-tRNA through the ribosome^18^. A similar indispensable role is assumed for the *T. brucei* RPS12 because the inactivation of RNA editing leads to the reduction of the 45S SSU-related complex and the 80S mRNA-bound monosome^7^. Furthermore, while only a few (cytochrome b, cytochrome oxidase subunit 1, F_o_F_1_-ATP synthase subunit A6) of the highly hydrophobic proteins translated from the maxicircle transcripts can be reliably detected in just procyclic form parasites^19,20^, the disruption of RNA editing severely impairs mitochondrial translation. Therefore, it is proposed that RPS12 acts as a functional link between RNA editing and translation in *T. brucei*^7^.

Remarkably, due to their complex mt gene expression, several hundreds of proteins are required to synthesize the only two mt encoded proteins (RPS12 and F_o_F_1_-ATPase subunit A6) deemed to be essential for the extracellular pathogen to reside in the bloodstream of its mammalian host^7,21^. While the insect stage (procyclic form, PF) of the parasite depends on the oxidative phosphorylation pathway to generate sufficient quantities of cellular ATP, the bloodstream form (BF) exploits the high glucose content of its surroundings to synthesize ATP through glycolysis^22^. This bioenergetics adaptation to the varied nutrients available throughout its life cycle results in a dramatic remodeling of the singular mitochondrion. The tubular BF mitochondrion lacks a functional cytochrome-mediated respiratory chain and thus respires through the trypanosome alternative oxidase^23,24^. Therefore, the F_o_F_1_-ATP synthase reverses direction and hydrolyzes ATP to pump protons into the mt inner membrane space, maintaining the essential mt membrane potential (∆ψ_m_)^25^. This rotary molecular machine is comprised of a catalytic F_1_ moiety and the membrane embedded F_o_ domain that contains the proton pore. Proton translocation occurs via interactions between the F_o_ c-ring and the only mt encoded component of F_o_F_1_-ATPase, subunit A6. The A6 transcript of this maxicircle gene is pan-edited in both life cycle stages, but it is only in the reduced BF mitochondrion that a single point mutation in the γ subunit of the F_1_ central stalk permits the parasite to become mt DNA independent^21^. Naturally occurring dyskinetoplastic (Dk) trypanosomes that lack a complete mt genome^26^, have also obtained compensatory mutations that allow the parasite to escape their dependency on the proton pumping function of the F_o_F_1_-ATPase. While the hydrolytic function of this enzyme is still essential, there is a greater reliance on the ADP/ATP carrier (TbAAC)^27,28^ to not only provide ATP substrate, but to also maintain the ∆ψ_m_ through the electrogenic exchange of cytosolic ATP^4−^ for mt ADP^3−^^21,29^.

Mt translation is a complex process that can be divided into four steps: initiation, elongation, termination and ribosome recycling. Here we focus on translation termination, which occurs when a mt release factor (RF), containing two decoding motifs (α−5 helix and PXT), determines that a mRNA stop codon occupies the ribosomal A site^30,31^. Upon recognition, the conserved GGQ loop of the RF shifts into the LSU peptidyl-transferase center (PTC) and hydrolyzes the ester bond between the tRNA in the peptidyl tRNA site (P site) and the terminal amino acid of the nascent peptide, thus releasing the protein from the translational apparatus^32,33^. Most mitochondrial translation systems have a reduced number of termination codons (UAA and UAG) that can be recognized by a single mt release factor (yeast – Mrf1)^34^. The depletion of Mrf1 in *S. cerevisiae*, a petite-positive yeast, results in respiratory dysfunction and rapid mt genomic instability, indicating that Mrf1 is essential for mitochondrial translation^35^. However, the depletion of this ortholog in either mammalian cells or *S. pombe*, a petite-negative yeast, only leads to a partial respiratory defect because mt protein synthesis is not completely eliminated^34^. This outcome suggests that another member of the RF family can compensate for the loss of the Mrf1. Indeed, while the depletion of the peptidyl-tRNA hydrolase, Pth4 in *S. pombe*, does not result in any phenotype, its ablation significantly exacerbates the Δmrf1 phenotype^36^. Pth4 retains the highly conserved GGQ motif, but it is smaller than standard RFs because it lacks the codon recognition domains^37^. Instead, it has an unstructured basic residue-rich tail at the C-terminus that is required for ribosomal binding. Unlike other members of the RF family that transiently interact with the ribosomal A site, Pth4 is an integral member of the LSU^37,38^. Therefore, it has been proposed that Pth4 acts as a codon-independent, mitoribosome-dependent RF that can rescue stalled ribosomes.

Mt translation is required for BF *T. brucei* viability^39^, but it presumably serves only to synthesize the essential F_o_F_1_-ATPase subunit A6. While TbMrf1 is indispensable in PF *T. brucei*^40^, where translation of most of the mt genome is required to generate sufficient ATP via an active oxidative phosphorylation pathway, we determined that the BF parasites grown in culture are able to adapt to life without TbMrf1.

## Results

### Cultured BF T. brucei tolerate the loss of TbMrf1, displaying only a mild growth phenotype

**Figure 1:**
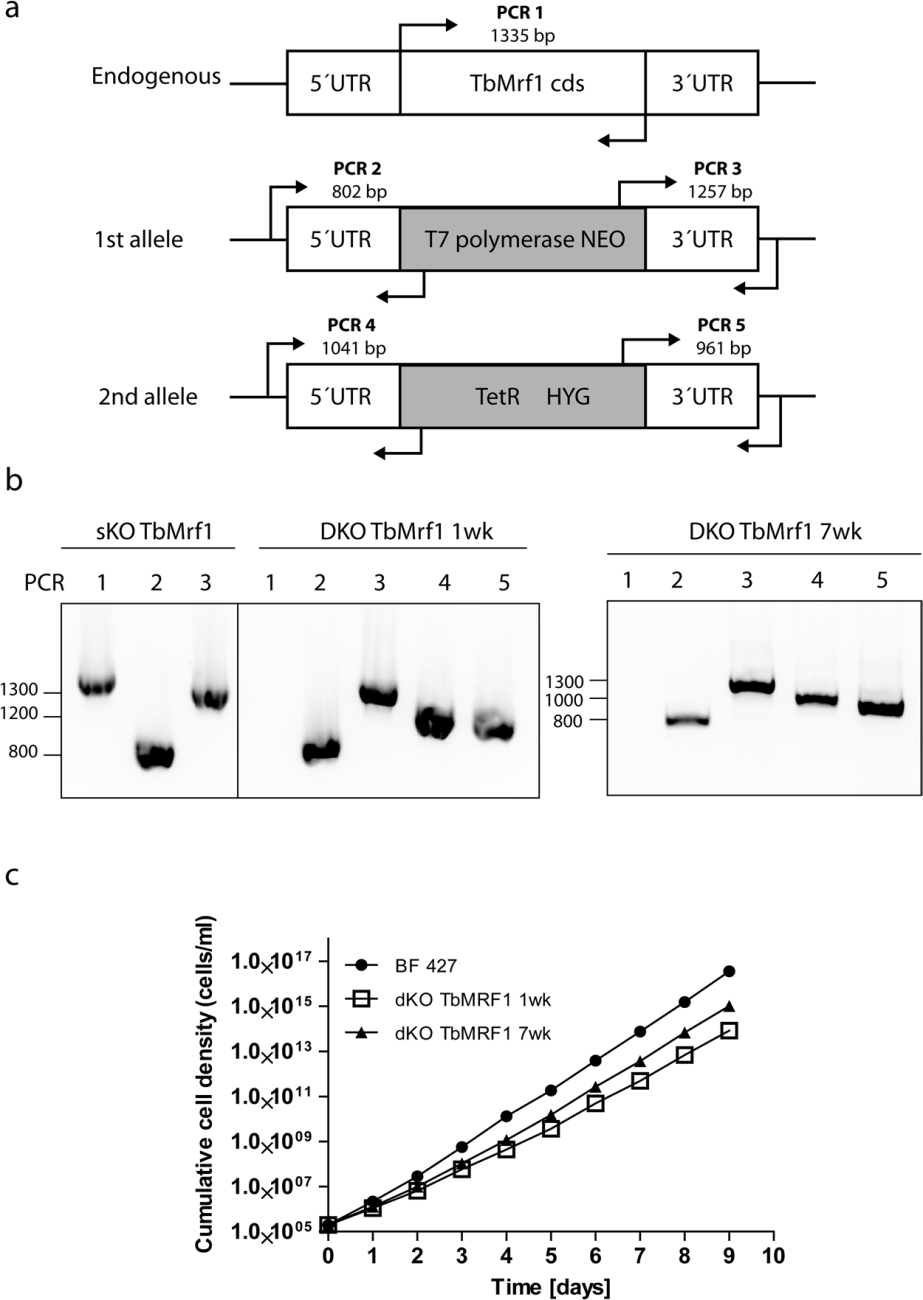
*T. brucei* BF dKO TbMrf1 parasites are viable in culture. (**a**) Schematic representation of the TbMrf1 gene knockout strategy based on homology recombination. To verify the correct integration of both allelic exchange cassettes by PCR, different primers sets were used to amplify PCR amplicons 1 to 5, with the expected sizes indicated. (**b**) PCR verification for the first TbMrf1 allelic replacement (sKO TbMrf1) and for the elimination of both TbMrf1 alleles in a dKO cell line grown in culture for one or seven weeks (dKO TbMrf1 1wk and 7wk). (**c**) Growth of dKO_TbMRF1 1wk and 7wk cell lines was monitored daily for nine days and compared to parental BF 427 cells. Cumulative cell density was plotted on a log scale.

Due to the limitations of RNAi to wholly silence a gene product, we eliminated both alleles of TbMrf1 (Tb927.03.1070) by homologous recombination. The generation of a viable double knockout TbMrf1 (dKO TbMrf1) cell line indicates that this release factor is not essential for the reduced mitochondrion of BF *T. brucei*. Lacking a TbMrf1 antibody, we verified the null cells by PCR analysis, incorporating primers designed to bridge each of the integration sites of the selectable markers that replaced both alleles (Fig. 1a). The single knockout (sKO) TbMrf1 cell line contained amplicons of the expected size for the TbMrf1 coding sequence (cds), as well as the 5′ and 3′ integration sites for the T7 polymerase/neomycin cassette (Fig. 1b). Noticeably, the TbMrf1 cds amplicon is not detected in the dKO cell line, while the replacement of both alleles was confirmed. Even though the dKO TbMrf1 cell line is certainly viable, it does have a reduced doubling time compared to the parental cell line (Fig. 1c, Table 1). Furthermore, we observed that dKO Mrf1 cells maintained in culture for 7 weeks had a modified rate of cell proliferation that reverted back towards the values of the wild-type (WT) BF 427 cells. Since these parasites appeared better adjusted to life without TbMrf1, we included this dKO TbMrf1 7wk cell line throughout our phenotypic analyses so they could be compared with the dKO TbMrf1 1wk cell line, which were the first cells to arise during the selection of transfected cells.

**Table 1:** Doubling time of *T. brucei* BF 427, dKO TbMrf1 1wk and 7wk cell lines.

Cell line	Doubling time [hours]
BF 427	5.5 ± 0.3
dKO TbMrf1 1wk	7.1 ± 0.7
dKO TbMrf 1 7wk	6.4 ± 0.6

The values represent the average and SD of three independent experiments.

The P value of an unpaired Student’s t-test comparing the doubling time values for BF 427 and dKO TbMrf1 1wk, BF 427 and dKO TbMrf1 7wk and dKO TbMrf1 1wk and 7wk are as follows: p < 0.001, p = 0.001 and p = 0.02, respectively.

### The absence of TbMrf1 significantly alters the structural integrity of the protein complexes containing mt encoded subunits

**Figure 2:**
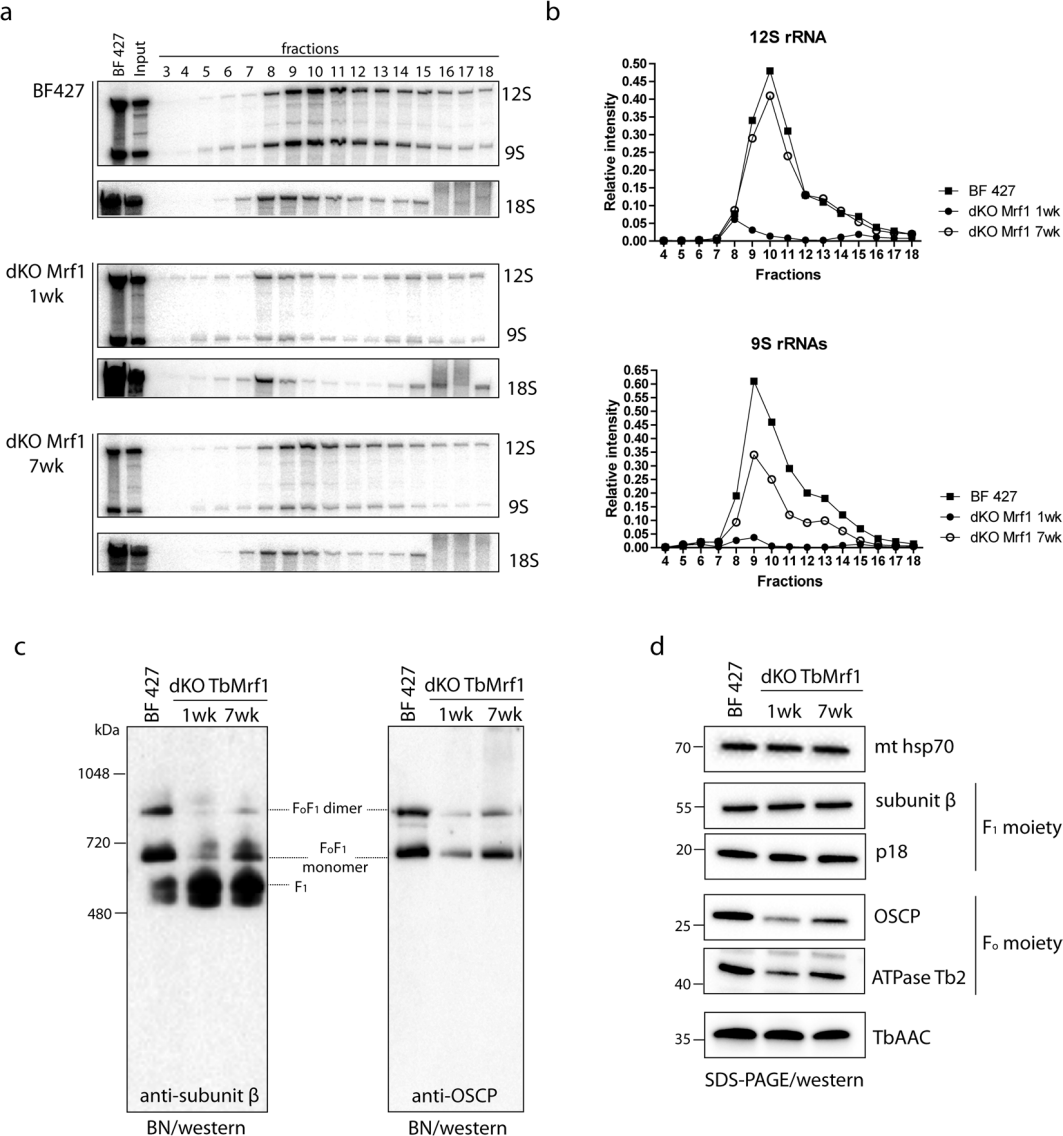
Loss of TbMrf1 affects the structural integrity of protein complexes that contain mt encoded subunits. (**a**) The sedimentation pattern of 12S and 9S rRNAs in BF 427 and dKO TbMrf1 1wk and 7wk cell lines. Whole cell lysates from 5 × 10^8^ parasites were resolved on a 10–30% glycerol gradient. RNA was extracted from each fraction and separated on 5% polyacrylamide/8 M urea gels that were blotted and probed for 12S and 9S mitochondrial rRNAs and 18S cytosolic rRNA (sedimentation control). BF 427–8 μg of total RNA from BF 427. Input – 3 μg of total RNA isolated from the remaining material that was loaded on a gradient. (**b**) After normalization to BF 427 RNA, the relative intensities of 9S and 12S rRNA signals from each sample were plotted. (**c**) The native F_1_- and F_o_F_1_-ATPase complexes were visualized using light blue native electrophoresis. Purified mitochondria from BF 427 and dKO TbMrf1 1wk and 7wk cultures were lysed with dodecyl maltoside, fractionated on 3–12% BisTris gel and blotted on a PVDF membrane. The F_1_-ATPase (F_1_) and the F_o_F_1_-ATPase monomer and dimer were all visualized using specific polyclonal antibodies against F_1_-ATPase subunit β and F_o_-ATPase subunit OSCP. (**d**) SDS-PAGE Western blot analyses of the same mitochondrial lysates as in (**c**). The steady state abundance of mt hsp70, TbAAC, F_1_-ATPase subunits β and p18 and F_o_-ATPase subunits OSCP and ATPaseTb2 were determined using specific antibodies.

While de novo mt synthesis of cytochrome b, cytochrome oxidase subunit 1 and F_o_F_1_-ATP synthase subunit A6 can be observed in PF parasites, none of the highly hydrophobic mt encoded proteins have ever been detected in the BF mitochondrion^20^. Therefore, to circumvent the dearth of direct assays to measure mt translation in the dKO TbMrf1 cell line, we opted to investigate the structural integrity of the mitoribosomes and F_o_F_1_-ATPase. Both of these large molecular complexes contain just a single gene product (RPS12 and A6, respectively) that is synthesized from the mt genome after extensive post-transcriptional editing in BF parasites. To determine how the loss of TbMrf1 affects the mitoribosomes, equal numbers of cells from the parental and dKO TbMrf1 cultures were lysed with 1% Nonidet NP40 and fractionated on a 10–30% glycerol gradient. Total RNA isolated from equal volumes of each fraction was resolved on a 5% polyacrylamide/8 M urea gel and visualized with 9S and 12S rRNA probes (Fig. 2a). To verify that each glycerol gradient created on the Gradient Master from BioComp Instruments produced reproducible sedimentation profiles, the total RNA samples from each fraction were also analyzed with a cytosolic LSU 18S rRNA probe. To compare the amount of rRNA in each fraction between the various cell lines, 8 ug of BF 427 total RNA was included for each northern blot. After normalization to this input, the relative intensity of 9S and 12S rRNA was plotted (Fig. 2b). The drastic decrease in both rRNA molecules throughout the gradient fractions of the dKO TbMrf1 1wk cell line indicates that the translational apparatus has been impaired, as both the LSU and SSU become unstable. Interestingly, it appears that the sedimentation profile has generally been restored in the dKO TbMrf1 7wk cell line, even though the recovery of the 9S rRNA trails that of the 12S rRNA.

The supramolecular organization of the F_o_F_1_-ATPase can be visualized when hypotonically isolated mitochondria are lysed with n-dodecyl-β-D-maltoside (DDM), resolved by light blue native (BN) electrophoresis and then transferred to a PVDF blot that is probed with serum that recognizes components of either the F_o_ or F_1_ moieties. In the absence of subunit A6, the higher molecular weight structures of the intact F_o_F_1_-ATPase are destabilized under the aforementioned conditions^41,42^. Strikingly, the dKO TbMrf1 1wk F_o_F_1_-ATPase monomers and oligomers are drastically reduced compared to the parental enzyme, whereas F_1_ accumulates (Fig. 2c). In addition, there is a recovery of the ATPase monomers in the dKO TbMrf1 7wk cell line. To demonstrate equal loading between the different cell lines, the same DDM-lysed mitochondria were also resolved by SDS-PAGE and transferred to membranes that were immunodecorated with a mtHSP70 monoclonal antibody (Fig. 2d). Furthermore, the decreased steady state expression of specific F_o_ subunits (Fig. 2d) in these denatured samples correlates with the loss of ATPase monomers and dimers visualized by BN westerns. On the other hand, components of the F_1_ catalytic moiety and the TbAAC transporter responsible for supplying this enzyme with its ATP substrate remain unchanged.

### Despite reduced ATPase monomers, dKO TbMrf1 cells still rely on the proton pumping activity of the remaining intact enzymes

**Figure 3:**
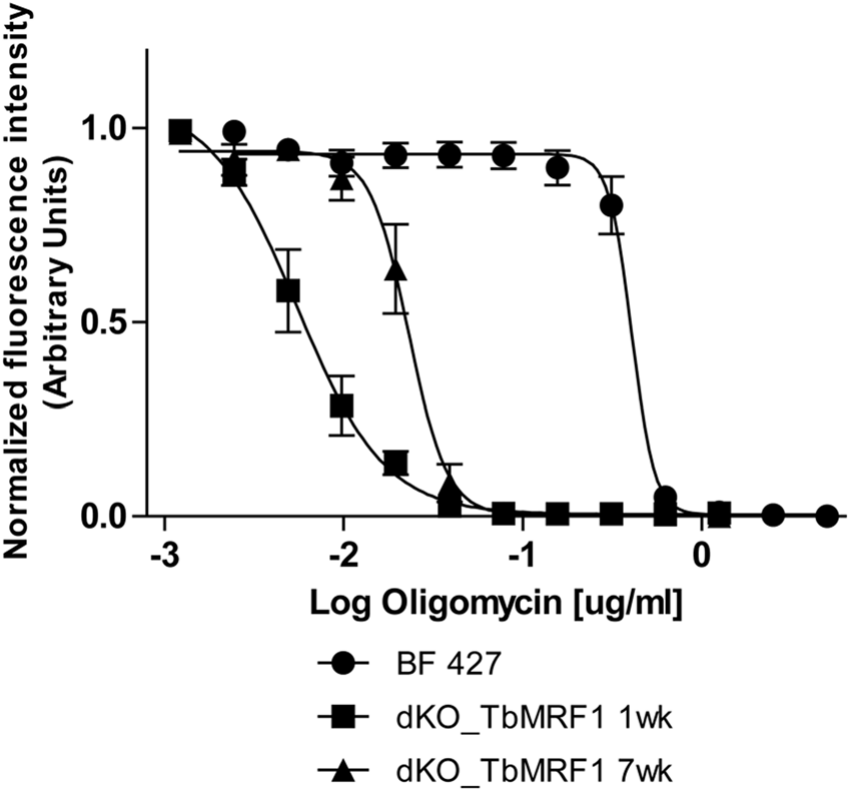
dKO TbMrf1 1wk and 7wk cell lines are significantly more sensitive to oligomycin than BF 427 cells. The oligomycin sensitivity of BF427, dKO TbMrf1 1wk and 7wk cells was determined by an Alamar Blue assay. The oligomycin dose-response curves were calculated using GraphPad Prism. Error bars represent the standard deviation calculated from three independent experimental replicates.

While the stability of the F_o_F_1_-ATPase monomers and oligomers is greatly reduced in the dKO TbMrf1 1wk culture, these structures are still detected. To determine if these DDM-resistant complexes contribute to cell viability, an Alamar Blue assay was performed to calculate the EC_50_ of oligomycin. This potent inhibitor binds at the interface of subunit c and A6, thereby blocking the proton translocation of intact F_o_F_1_-ATPase^43^. Even though BF 427 parasites are highly sensitive to oligomycin because they depend on the F_o_F_1_-ATPase activity to pump protons that maintain the ∆ψ_m_, the dKO TbMrf1 1wk cells are several orders of magnitude more sensitive to this antibiotic (Fig. 3, Table 2). Once again, the dKO TbMrf1 7wk culture demonstrates an intermediate phenotype as the oligomycin EC_50_ increases towards the values measured in the parental cell line.

**Table 2:** EC_50_ values for oligomycin, carboxyatractyloside and acriflavin for BF 427, dKO TbMrf1 1wk and 7wk and dKO TbMrf1 + V5 TbPth4 cells that were either noninduced (−tet) or induced with tetracycline (+tet) for 2 days.

Cell line	Oligomycin [ug/ml]	Carboxyatracty-loside [mM]	Acriflavin [nM]
BF 427	0.403 ± 0.03	>0.369	6.02 ± 0.52
dKO TbMRF1 1wk	0.006 ± 0.006	0.017 ± 0.02	2.68 ± 0.26
dKO TbMRF1 7wk	0.022 ± 0.005	0.076 ± 0.02	3.55 ± 0.17
cKO TbMrf1 7wk (+tet)	0.13 ± 0.05	0.125 ± 0.01	n.d.
*T. evansi* Antat 3/3	>2.657	0.009 ± 0.002	n.d.
dKO TbMRF1 7wk + V5 TbPth4 (– tet)	0.013 ± 0.03	0.027 ± 0.05	n.d.
dKO TbMRF1 7wk + V5 TbPth4 (+tet)	0.027 ± 0.01	0.06 ± 0.09	n.d.

n.d. – not determined.

The values represent the average and SEM of three independent experimental replicates.

### Destabilization of intact F_o_F_1_-ATPase complexes only partially diminishes the ∆ψ_m_

**Figure 4:**
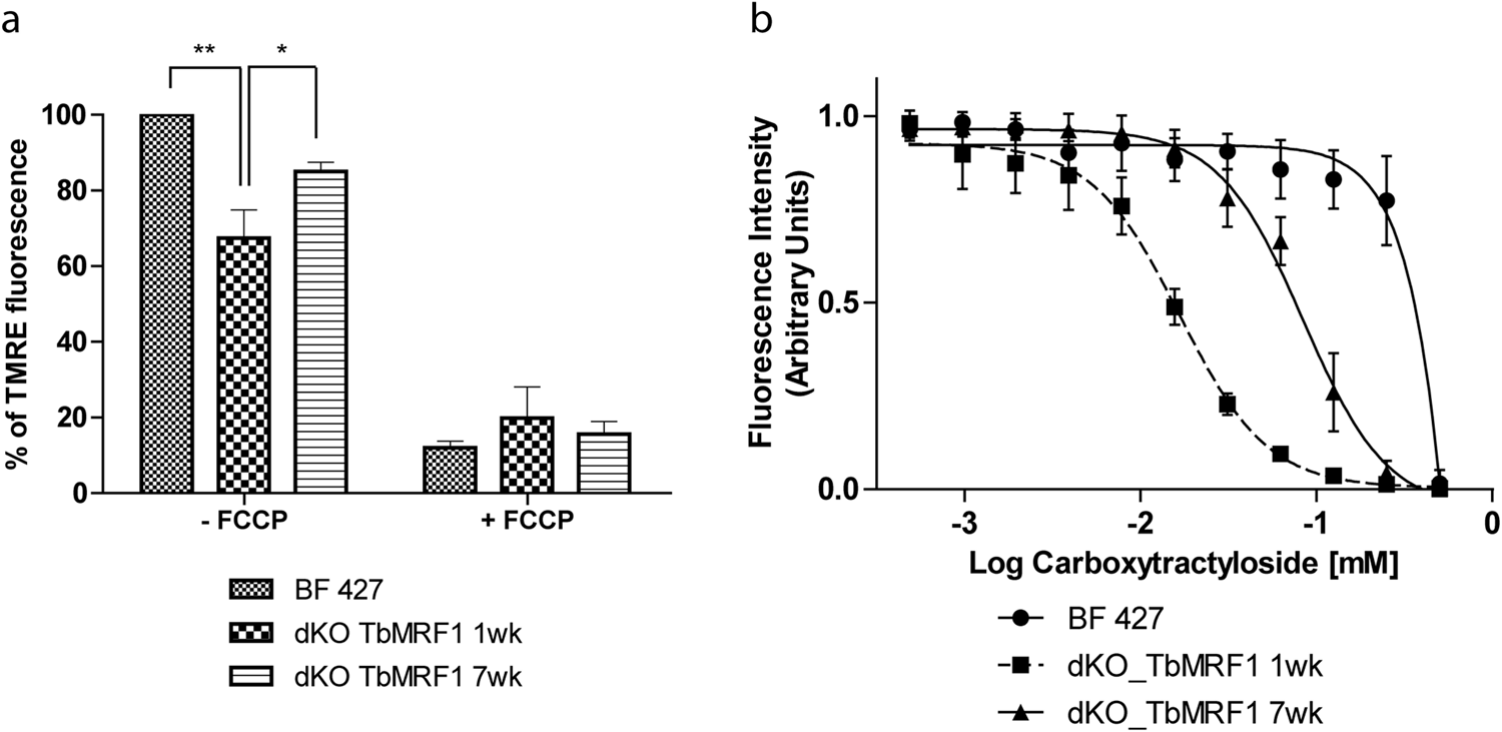
∆ψm is only partially diminished in dKO TbMrf1 1wk and 7wk cells. (**a**) The ∆ψ_m_ of cells stained with TMRE was measured by flow cytometry. The median fluorescence for each sample is depicted on the y-axis of the column graph. The results are means ± s.d. (n = 5). **p < 0.002, *p < 0.02, Student’s t test. (**b**) Carboxyatractyloside sensitivity of BF 427 and dKO TbMrf1 1wk and 7wk cells was assessed using an Alamar Blue assay. The dose-response curves were calculated using GrapPad Prism. Error bars represent the standard deviation calculated from three independent experimental replicates.

In BF *T. brucei* mitochondria, the intact F_o_F_1_-ATPase hydrolyzes ATP and pumps protons into the mt intermembrane space to maintain the essential ∆ψ_m_. Since the absence of TbMrf1 leads to the partial destabilization of F_o_F_1_-ATPase monomers and dimers, the relative ∆ψ_m_ was determined by staining these cell lines with TMRE and measuring the fluorescence produced from the ∆ψ_m_ dependent probe. Compared to BF 427 cells, the ∆ψ_m_ was diminished ~35% in the dKO TbMrf1 1wk cells (Fig. 4a), which most likely contributed to the mild growth phenotype seen in these cells. Furthermore, the ∆ψ_m_ of the dKO TbMrf1 7wk cells returned to within ~15% of BF 427 values, which correlates with the increased stability observed in the F_o_F_1_-ATPase monomers in these parasites (Fig. 2c).

While there were dramatically fewer intact F_o_F_1_-ATPases to maintain the ∆ψ_m_ in the dKO TbMrf1 1wk cell line (Fig. 2c), the depolarization of the inner mt membrane was not as drastic as when a subunit of the catalytic F_1_ moiety is depleted^25,44^. Therefore, we hypothesized that the mt bioenergetics of dKO TbMrf1 1wk cells might more closely resemble Dk trypanosomes, where the dependence on the electrogenic exchange of cytosolic ATP^4-^ for mt ADP^3−^ by TbAAC becomes magnified. To assess this possibility, we employed an Alamar Blue assay to measure the EC_50_ of carboxyatractyloside (CATR), an inhibitor of AAC, in BF 427 cells, dyskinetoplastic *T. evansi* and the dKO TbMrf1 cell lines. While there is no significant change in the steady state levels of TbAAC expression when the parasite lacks TbMrf1 (Fig. 2d), the sensitivity to CATR is drastically increased in the dKO TbMrf1 1wk cells to almost the same levels observed in *T. evansi* (Fig. 4b, Table 2). Furthermore, the intermediate CATR EC_50_ values measured in the dKO TbMrf1 7wk cells correlates with the observed increased levels of intact F_o_F_1_-ATPase monomers in these parasites.

Dk trypanosomes are able to lose their mt genomes because they contain compensatory mutations within specific F_1_-ATPase subunits. However, when we sequenced the γ subunit from dKO TbMf1 7wk culture, no mutations including the ones known for Dk cells^26^ were observed (data not shown). Furthermore, the completed genome of the dyskinetoplastic *T. evansi* strain STIB805 revealed that there are only two notable amino acid substitutions in the α (G510C) and β (N395S) subunits^45^, but neither one was detected in our sequencing data from the dKO TbMrf1 7wk cell line. In fact, unlike yeast Mrf1 mutants that display a rapid lost of mt DNA, the DAPI staining of fixed *T. brucei* lacking TbMrf1 did not result in cells without kinetoplast DNA (Supplementary Fig. S1). Furthermore, the dKO TbMrf1 cell lines are slightly more sensitive than BF 427 cells to acriflavin, a lethal DNA intercalator that predominantly accumulates in the mt DNA (Table 2). Finally, the calculated oligomycin EC_50_ value of the dyskinetoplastic *T. evansi* (Table 2) indicates that unlike the dKO TbMrf11wk cell line, these parasites are even less sensitive to oligomycin than BF 427. Altogether, this would suggest that the dKO TbMrf1 mutants are still dependent on their mt genome expression.

To confirm that the observed phenotypes measured were truly due to the loss of TbMrf1, we generated a conditional knockout (cKO) TbMrf1 7wk cell line with heterologous expression of a C-terminally V5-tagged TbMrf1 that was verified by western blot analysis with a V5 antibody (Supplementary Fig. S2a). This cell line demonstrated that despite the epitope tag, tetracycline induced ectopic TbMrf1 expression largely restored the oligomycin and carboxyatracrtyloside EC_50_ values to BF 427 levels (Supplementary Fig. S2b, Table 2).

### TbMrf1 deficient parasites are avirulent in the mouse model

**Figure 5:**
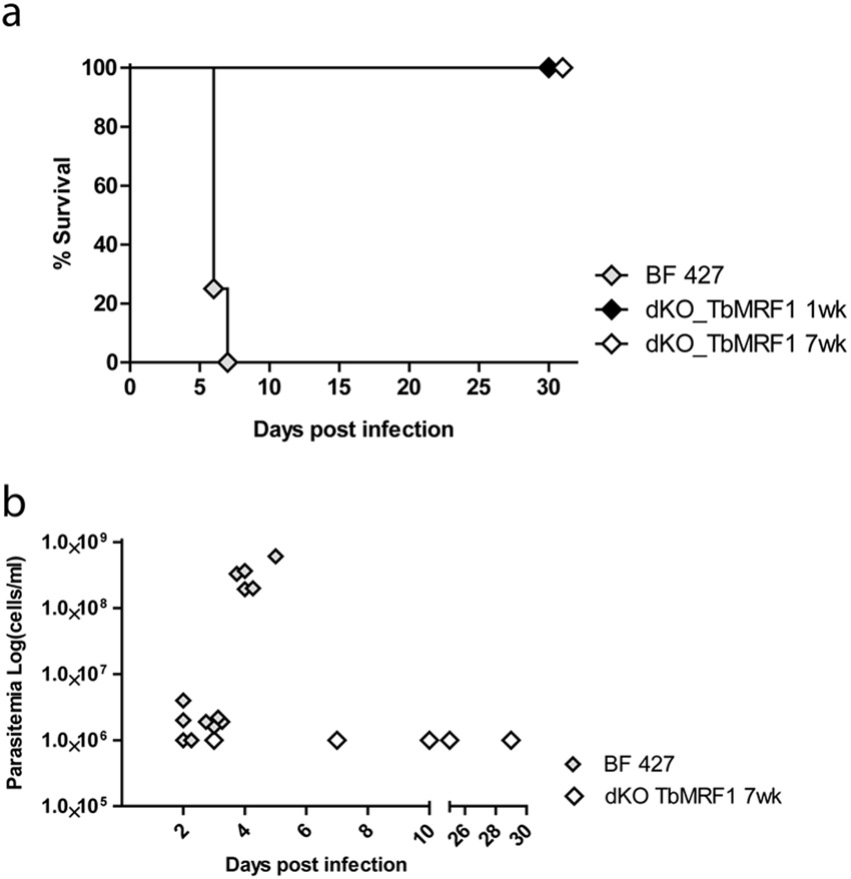
dKO TbMrf1 parasites are avirulent in a mouse model. (**a**) Groups of five female BALB/c mice were intraperitoneally infected with 1 × 10^5^ BF 427 and dKO TbMrf1 1wk and 7wk trypanosomes. The survival rate of infected mice was monitored for 30 days. (**b**) Mouse parasitemia levels were calculated daily post-infection.

While dKO TbMrf1 cell lines are able to cope with a significant loss of intact F_o_F_1_-ATPase complexes and remain viable when grown in culture, we questioned whether these parasites would be virulent when introduced into an animal model with an active immune system. Therefore, we infected 3 groups of 5 BALB/c mice with 1 × 10^5^ BF 427 or dKO TbMrf1 1wk or 7wk parasites. The survival rate was monitored and parasitemia levels were calculated from blood samples obtained via tail pricks. While none of the mice infected with BF 427 trypanosomes survived past day 6, neither set of mice infected with either dKO TbMrf1cell line succumbed to disease after 30 days (Fig. 5a). This discrepancy was further illustrated in the parasitemia levels, where BF 427 cells were quickly able to reach lethal levels (>1 × 10^8^ trypanosomes/ ml blood), while only very low levels of dKO TbMrf1parasites were sporadically detected throughout the length of the experiment (Fig. 5b). Thus, while convenient, measuring the growth phenotypes of genetically modified trypanosomes in culture does not always depict the true importance of a protein function, especially in the case of gene products that can potentially disrupt bioenergetic processes of the cell.

### Overexpression of release factor TbPth4 partially alleviates the dKO TbMrf1 phenotypes

**Figure 6:**
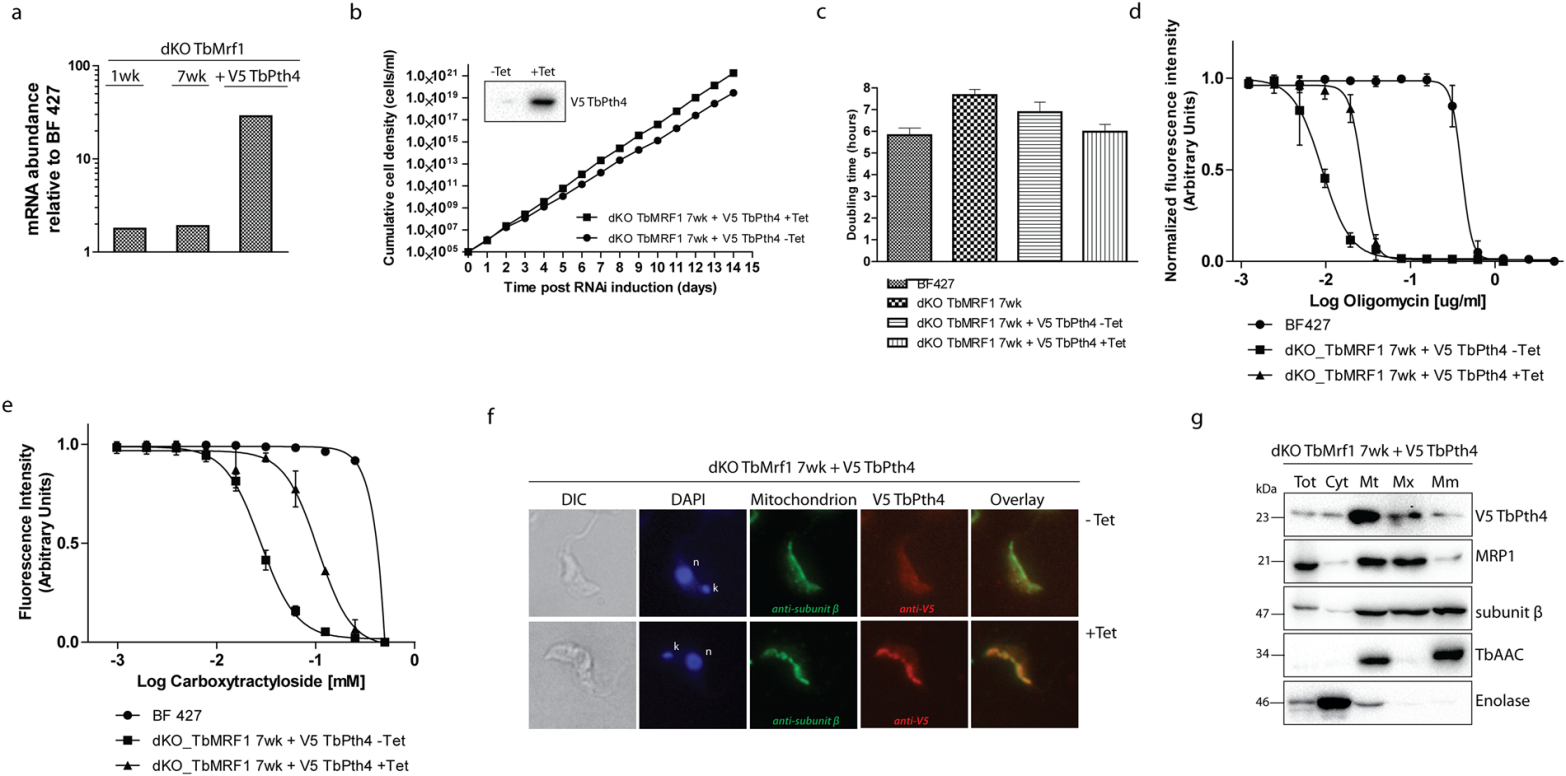
Overexpression of mitochondrially localized TbPth4 abates the dKO TbMrf1 phenotype. (**a**) RT-qPCR analysis of TbPth4 transcript levels in dKO TbMrf1 1wk, 7wk and dKO TbMrf1 + V5 TbPth4 cells compared to BF 427 trypanosomes. The relative changes in transcript abundance were plotted on a log scale. Transcript levels of β-tubulin were used as internal controls. (**b**) Growth curves spanning 14 days were generated for tetracycline induced (+tet) and noninduced (-tet) dKO TbMrf1 + V5 TbPth4 cells. The figure is representative of three independent experimental tetracycline inductions. Inset: Whole cell lysates from cultures either noninduced (-tet) or induced ( + tet) with tetracycline for 48 hours were probed with a specific anti-V5 antibody. (**c**) Plot of doubling times calculated from the linear growth of BF 427, dKO TbMrf1 7wk and tetracycline induced (+tet) and noninduced (-tet) dKO TbMrf1 + V5 TbPth4 cells. (**d**) & (**e**) Alamar Blue assays determined either the oligomycin (**c**) or carboxyatractyloside (**d**) sensitivity of induced (+tet) and noninduced (-tet) dKO TbMrf1 + V5 TbPth4 cells compared to BF 427 cells. Error bars represent the standard deviations calculated from three independent experimental replicates. (**f**) Immunofluorescence assays verify that V5-tagged TbPth4 is targeted to the mitochondrion in BF dKO TbMrf1 + V5 TbPth4 cells induced for 48 hours (+Tet). The ectopic TbPth4 was visualized with a Texas Red-conjugated secondary antibody that recognizes a primary monoclonal anti-V5 antibody. Noninduced (-Tet) BF BF dKO TbMrf1 + V5 TbPth4 cells were included as a control, while the single tubular mitochondrion was visualized with a fluorescein isothiocyanate (FITC)-conjugated secondary antibody that recognizes a polyclonal primary antibody detecting F_1_-ATPase subunit β. The DNA contents were visualized using DAPI (4,6-diamidino-2-phenylindole). The overall cell morphology is depicted in the differential interference contrast (DIC) microscopy images. (**g**) Subcellular localization of TbPth4 was determined in dKO TbMrf1 cells expressing V5-tagged TbPth4. Harvested parasites (Tot) were lysed under hypotonic conditions to obtain cytosolic (Cyt) and mitochondrial (Mt) fractions. Mt pellets treated with Na_2_CO_3_ underwent differential centrifugation to produce matrix (Mx) and mt membrane (Mm) fractions. Purified fractions were analyzed by Western blot with the following antibodies: anti-V5 (V5-TbPth4), anti-MRP1 (mt matrix), subunit β (mt matrix and membranes), anti-AAC (mt membranes), anti-enolase (cytosol).

The viability of the dKO TbMrf1 cell line in culture suggests the presence of another factor that is able to compensate for the loss of TbMrf1 and ensure some mt translation termination. In *S. pombe*, it has been demonstrated that the peptidyl-tRNA hydrolase Pth4 plays an overlapping role with Mrf1^36^. A protein BLAST search on TriTrypDB revealed that TbPth4 (Tb927.6.2500), which contains a release factor domain, is the most obvious *T. brucei* ortholog of the yeast protein as they share 36% similarity and 21% identity (MUSCLE alignment). To ascertain if the function of TbPth4 becomes more important when the mt translation appartus becomes impaired, we monitored its gene expression in the dKO TbMrf1 cell lines. Since we lack an antibody that recognizes TbPth4, we employed qPCR to analyze the levels of mRNA in the dKO TbMrf1 cell lines compared to BF 427. Using β-tubulin as an internal control, a very modest increase of TbPth4 mRNA was observed in the absence of TbMrf1 (Fig. 6a). However, compared to the considerable Pth4 mRNA expression observed in the tetracycline induced dKO TbMrf1 cell line overexpressing a V5-tagged ectopic TbPth4, this slight uptick may not translate into any biological significance in the dKO TbMrf1 parasites.

Since the overexpression of the *S. pombe* Pth4 helps to alleviate the phenotypes induced by the loss of Mrf1^36^, we explored if the same could be true in *T. brucei*. While the dKO TbMrf1 1wk cell line demonstrates the largest phenotypic changes compared to BF 427, we have demonstrated that the severity of these measured outcomes reduces over time as the cells are maintained in culture. Therefore, due to the length of time involved in selecting transfected *T. brucei* and verifying positive clonal cell lines, it was not feasible to generate the ectopic V5-tagged TbPth4 in the dKO TbMrf1 1wk cell line. However, when the V5-tagged TbPth4 is overexpressed in the dKO TbMrf1 7wk background, the doubling time continues to return towards WT values (Fig. 6b,c). We also determined from additional Alamar Blue assays that the increased sensitivity to oligomycin and carboxyatractyloside observed in the dKO TbMrf1 cell lines is reduced when the ectopic TbPth4 is induced (Fig. 6d,e, Table 2).

Since TbPth4 has not been characterized previously, we analyzed the subcellular localization of the release factor. An immunoflourescence assay determined that the dKO TbMrf1 + V5 Pth4 is colocalized with the F_1_-ATPase subunit β in the mitochondrion (Fig. 6f). Furthermore, the tagged TbPth4 was tracked in a Western blot analysis of fractions generated from a carbonate extraction of enriched mitochondria isolated by hypotonic lysis of the parasite. The specificity of the resulting subcellular fractions was verified using enolase as a cytosolic marker; while MRP1, ATPase subunit β and TbAAC represented mt proteins with various propensities for either the mt inner membrane or the matrix. This analysis reveals that TbPth4 is largely localized in the mt matrix (Fig. 6g).

### Depletion of TbPth4 exacerbates the phenotypes identified in BF T. brucei lacking TbMrf1

**Figure 7:**
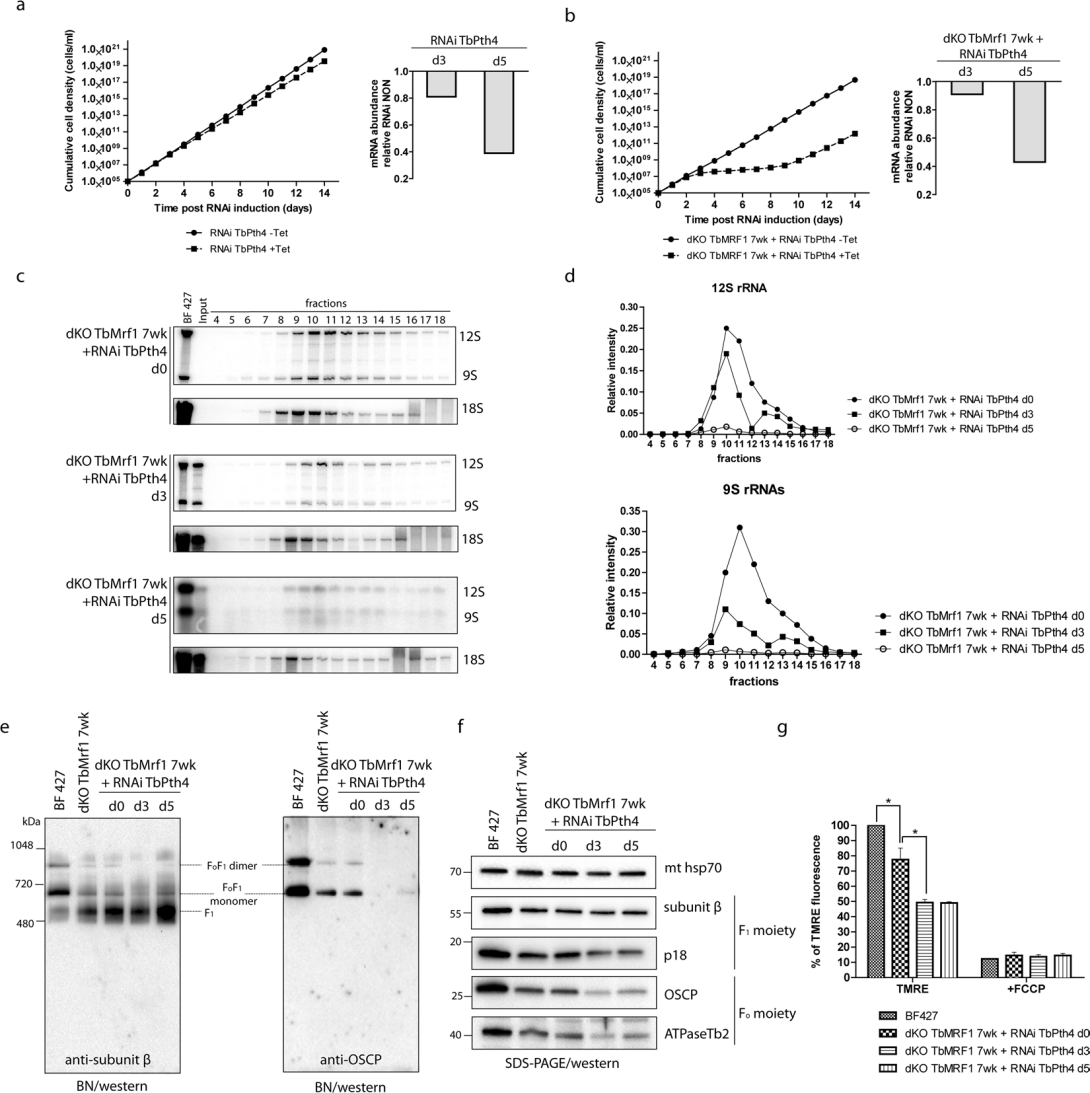
TbPth4 RNAi silencing in the dKO TbMrf1 background generates more severe phenotypes than the loss of TbMrf1 alone. (**a**,**b**) Growth curves of the noninduced (-tet) and induced (+tet) RNAi TbPth4 in either SM BF 427 (**a**) or dKO TbMrf1 cells (**b**) were measured for 14 days. Right panels: RT-qPCR analysis of the TbPth4 transcript levels after 3 (d3) or 5 (d5) days of tetracycline induction compared to noninduced RNAi cells. The relative changes in transcript abundance are plotted on a linear scale. β-tubulin transcript levels were used as internal controls. (**c**) The sedimentation profile of 12S and 9S rRNAs in dKO TbMrf1 + RNAi TbPth4 cells that were either never induced (d0) or induced with tetracycline for 3 (d3) or 5 (d5) days. BF427–8 μg of total RNA from BF 427. Input – 3 μg of total RNA isolated from the remaining material (if available) that was loaded on a gradient. (**d**) After normalization to a BF 427 RNA, the relative intensities of 9S and 12S rRNA signals from each sample were plotted. (**e**) The native F_1_- and F_o_F_1_-ATPase complexes were visualized using light blue native electrophoresis. dKO TbMrf1 7wk and dKO TbMrf1 + RNAi TbPth4 cells were either never induced (d0) or induced with tetracycline for 3 (d3) or 5 (d5) days. The F_1_-ATPase (F_1_) and the F_o_F_1_-ATPase monomer and dimer were all visualized using specific polyclonal antibodies against either F_1_-ATPase subunit β or F_o_-ATPase subunit OSCP. (**f**) SDS-PAGE Western blot analyses of the same mitochondrial lysates as in (**e**). The steady state abundance of mt hsp70, F_1_-ATPase subunits β and p18 and F_o_-ATPase subunits OSCP and ATPaseTb2 were determined using specific antibodies. (**g**) The ∆ψ_m_ of TMRE stained BF 427 and dKO TbMrf1 + RNAi TbPth4 cells that were either never induced (d0) or induced with tetracycline for 3 (d3) or 5 (d5) days was measured by flow cytometry. The median fluorescence for each sample is depicted on the y-axis of the column graph. The results are means ± s.d. (n = 5). *p < 0.02, Student’s t test.

To determine if TbPth4 is involved in the rescue of an impaired mt translation system missing TbMrf1, we depleted this release factor in both BF 427 single marker (SM) and dKO TbMrf1 7wk cells using a stem loop RNAi vector. While there was a very modest increase in the doubling time observed in the RNAi induced BF 427 SM cells (Fig. 7a), there was a striking growth phenotype when TbPth4 is abated in the dKO TbMrf1 7wk cell line (Fig. 7b). A qPCR analysis verified that the TbPth4 transcript is similarly reduced in both cell lines. Typical of the RNAi system in *T. brucei*, the dKO TbMrf1 + RNAi TbPth4 culture escaped the RNAi regulation around day 10 of tetracycline induction.

We further characterized the dKO TbMrf1 + RNAi TbPth4 cell line by once again observing what happens to the structural integrity of the mitoribosomes. Since the parental cell line is the dKO TbMrf1 7wk culture, the uniduced RNAi cells display a fairly normal and broad sedimentation profile of the 9S and 12S rRNA on a glycerol gradient (Fig. 7c,d). By day 3 of the TbPth4 RNAi induction, the levels of both rRNAs are reduced, with a greater impact on the RPS12 associated 9S SSU peak. There are also two distinct rRNA peaks that presumably represent the individual SSU and LSU subcomplexes (45S) and the higher S-value (80S) monosome fraction associated with mRNA. However, after depleting TbPth4 for 5 days, the 80S rRNA peak is largely reduced and all that remains is a diminished broad peak centered around the lower 45S fractions. These results would suggest that the partially restored WT-like mitoribosome profile observed in the dKO TbMrf1 7wk cell line has been eliminated with the loss of TbPth4.

To understand how an impeded mt translation system could result in a cytostatic dKO TbMrf1 + RNAi TbPth4 cell line, we observed the supramolecular organization of the F_o_F_1_-ATPase by probing Western blots generated from light blue native gels with serum that recognize distinct ATPase moieties. Unlike the dKO TbMrf1 7wk cells that had recovered some of the F_o_F_1_-ATPase monomers, the monomers and dimers of this proton pumping enzyme are virtually undetectable by day 3 and 5 of TbPth4 RNAi induction (Fig. 7e). This result is further exemplified by the steady-state Western analysis of these same DDM-lysed mitochondria resolved on SDS-PAGE, which demonstrate that the F_o_ subunit OSCP that couples the catalytic activity of the F_1_ region to the proton pumping activity is not visualized upon the induction of TbPth4 RNAi (Fig. 7f). With little to no intact F_o_F_1_-ATPase with a functional proton pore that can directly contribute to the ∆ψ_m_, the measured relative ∆ψ_m_ of the induced dKO TbMrf1 + RNAi TbPth4 cells drops to 50% of wildtype levels (Fig. 7g).

## Discussion

Despite the severe growth phenotype previously observed in cultured PF *T. brucei* depleted of TbMrf1^40^, we were able to generate *in vitro* BF parasites without this release factor. Confronted with an intriguing BF cell line that can tolerate the elimination of TbMrf1, but without any direct means of ascertaining how mt translation termination was affected, we chose to characterize mitochondrial phenotypes that could rationally be attributed to the loss of a release factor. In fact, it was indirect evidence that originally assigned the expected function of TbMrf1 when it was shown that oxidative phosphorylation (OXPHOS) is disturbed upon TbMrf1 RNAi induction^40^. Unfortunately, even this assay is impractical when working with BF trypanosomes, so we decided to look at the integrity of two essential complexes in the BF mitochondrion that contain a solitary mt encoded subunit. Indeed, this is not unprecedented as the stability and composition of the mitoribosomes and F_o_F_1_-ATPase has been extensively characterized when mt gene expression is impeded^7,42^.

With this rationale, we interpreted the significant disruption of intact mitoribosomes and F_o_F_1_-ATPases in the dKO TbMrf1 1wk cell line to indirectly indicate that mt translation has been impaired. The instability of the F_o_F_1_-ATPase monomer and dimers is most likely due to the abated protein expression of the F_o_ integral membrane subunit A6, which causes the complex to become more labile in the presence of detergent^41,42,46^. However, since the dKO TbMrf1 parasites are hypersensitive to oligomycin and thus still depend on the proton pumping function of the remaining intact F_o_F_1_-ATPase enzymes, we believe that mt translation is not completely blocked. This analysis is further supported by the observations that the dKO TbMrf1 cells retain some intact F_o_F_1_-ATPases, do not lose their mt DNA, display increased sensitivity to acriflavin and do not possess any of the required γ mutations previously catalogued in dyskinetoplastic trypanosomes^26^.

The ability of the dKO TbMrf1 1wk cells to continue to synthesize some amount of subunit A6 in the absence of TbMrf1 presumably allows the parasite to adapt its bioenergetics by relying more heavily on TbAAC to produce an electrogenic exchange of cytosolic ATP^4−^ for mt ADP^3−^, which can compensate for the decrease in intact F_o_F_1_-ATPase complexes containing a functioning proton pore. Indeed, it appears that there is a delicate equilibrium that is maintained between the amount of proton pumping F_o_F_1_-ATPase enzymes and the activity levels of TbAAC to maintain the ∆ψ_m_ as evidenced by the increased levels of intact F_o_F_1_-ATPase monomers in the dKO TbMrf1 7wk cells and the intermediate CATR EC_50_ values observed for these parasites.

This unique compensatory mechanism can occur in the BF trypanosoma mitochondrion because of the unusal dependence on the F_o_F_1_-ATPase to continually hydrolyze ATP to maintain the essential ∆ψ_m_ required for mt protein import^47^. However, this comes at a large energetic cost to the parasite. Therefore, it is pertinent to understand how much of a ∆ψ_m_ is needed to maintain sufficient protein and metabolite (e.g. pyruvate) import for key mt processes^48^. By thoroughly characterizing these dKO TbMrf1cell lines, we propose that *in vitro* BF trypanosoma cell cultures can tolerate a substantial decrease in their ∆ψ_m_ as long as it remains above a minimal threshold, which we predict is between 50–65% of WT ∆ψ_m_ values. While this allows the parasite to survive in culture, it is no longer a viable option when the *T. brucei* dKO TbMrf1 is introduced into an animal model, probably because the parasite has greater energetic requirements when it needs to evade the host’s immune system^49^. Under these conditions, even the dKO TbMrf1 7wk parasites, observed to possess 85% of the WT ∆ψ_m_ when grown *in vitro*, are unable to establish a lethal infection in a mouse.

Canonical translation termination occurs when Mrf1 recognizes a stop codon in the A site and hydrolyzes the ester bond between the P site tRNA and the nascent polypeptide. However, ribosomes can become stalled when they encounter truncated mRNA or transcripts that contain nonstop mutations or rare codons^50^. Pth4 is a yeast codon-independent release factor that is described as a versatile mitoribosome rescue factor that is involved in recycling these stalled ribosomes. The observed phenotypes in both the induced dKO TbMrf1 + V5 Pth4 and dKO TbMrf1 + RNAi TbPth4 cell lines provide tantalizing evidence that TbPth4 is able to recognize that the dKO TbMrf1 mitoribosome has become stalled at the UAG stop codon and is able to release the completed polypeptide to create a sufficient amount of RPS12 and subunit A6.

While Pth4 homologs are found throughout bacteria and eukaryotic mitochondria^36,37,51^, it appears that they probably function via different molecular mechanisms. For example, it has been demonstrated that upon RNAi depletion of the Pth4 mammalian ortholog (ICT1), cell viability is greatly affected as well as the stability of mt encoded subunits of OXPHOS complexes, in particular cytochrome c oxidase^37^. However, the deletion of Pth4 in *S. pombe* caused no reduction in cell proliferation even when grown on media containing only non-fermentable carbon sources. Similarly, the silencing of TbPth4 in BF 427 *T. brucei* did not generate a significant growth effect.

It is currently unclear how this ribosome integrated release factor is able to gain access to the distant PTC and become catalytically active. The LSU bound ICT1 may be restricted to an architectural role, while soluble forms of the protein are able to bind the mitoribosomal A site and perform peptidyl-tRNA hydrolysis^50^. While no free pool of the ICT1 has yet to be detected in the mammalian mt matrix, ectopic expression of TbPth4 in *T. brucei* is prodominantly localized in the soluble mt fraction. This would suggest that the mechanism of TbPth4 will be more similar to yeast, which also has a fraction of the release factor that is unassociated with the mitoribosome. The overexpression of TbPth4 in the dKO TbMrf1 background likely increased the overall amount of the protein, most of which probably contributed to a pool of unbound release factor that was available to bind the empty A site of stalled mitoribosomes. However, this only produced mild decreases in the observed dKO TbMrf1 phenotypes, suggesting that this emergency release factor is not overly efficient at rescuing stalled ribosomes. This would perhaps explain why TbPth4 was not able to replace the loss of TbMrf1 in the procyclic stage of the parasite, where the bioenergetic needs of the active mitochondrion requires robust translation of nearly all 18 mt encoded transcripts. Additional studies are being pursued to determine the molecular mechanism in which TbPth4, a release factor without a codon recognition domain, is able to terminate translation at a stop codon.

## Methods

### Transgenic T. brucei cell cultures

The bloodstream form *T. brucei brucei* Lister 427 strain (information about the identity and genealogy of this strain is available at http://tryps.rockefeller.edu) and the dyskinetoplastic strain *T. b. evansi* Antat 3/3^52^ were cultured in HMI-9 media containing 10% FBS and incubated in a 37 °C incubator with 5% CO_2_. The BF 427 single marker (SM) cell line, constitutively expressing the ectopic copy of T7 RNA polymerase and tetracycline repressor, was used for the tetracycline inducible expression of dsRNA and V5-tagged proteins^53^. This expression system was induced by the addition of 1 μg/ml of tetracycline into the media. Cell densities were monitored using the Z2 Cell Counter (Beckman Coulter Inc.) and maintained in the mid-log phase (between 1 × 10^5^ to 1 × 10^6^ cells/ml). Growth curves depicting the cumulative cell number of the cultures were calculated from the measured cell densities that were adjusted by the dilution factor needed to seed the cultures at 1 × 10^5^ cells/ml each day.

The generation of a TbMrf1 double knockout (dKO) cell line involved replacing both alleles of the *TbMrf1* gene (Tb927.3.1070) with either a neomycin or hygromycin resistance gene cassette derived from the pLEW13 and pLEW90 vectors, respectively^53^. To direct the homologous recombination to the TbMrf1 alleles, these knockout (KO) cassettes were flanked by short sequences of the *TbMrf1* 5′ (469 nt) and 3′ (540nt) untranslated region (UTR) that were identified with TritrypDB (version Tb927_3_V5.1). These UTR fragments were PCR amplified from BF 427 genomic DNA (gDNA) with either the 5′UTR_fw&rv or the 3′UTR_fw&rv primer pairs (Supplementary Table S1). The amplicons were then digested either with NotI and MluI endonucleases (5′ UTR) or XbaI and StuI (3′ UTR) before sequentially being ligated into the neomycin-resistance cassette containing the T7 polymerase gene. The final pLEW13_TbMrf1_3′/5′UTRs_neomycin construct was linearized with NotI and electroporated with human T cell nucleofactor solution (AMAXA) into BF 427 to generate a single knockout (sKO) cell line. To create near-clonal cell lines, the transfected cells were serially diluted after 16 hours of recovery and selected with 2.5 μg/ml neomycin. The gDNA from stable transgenic cell lines that arose after ~2 weeks of selection was isolated using the GenElute Mammalian Genomic DNA Miniprep Kits from Sigma. The correct integration of the sKO cassette into the *TbMrf1* allele was verified by PCR with the following primer pairs that bridge either the 5′ or 3′ integration sites: 5′UTR_ext_fw and sKO_rv; sKO_fw and 3′UTR_ext_rv (Fig. 1a,b, Supplementary Table S1).

The hygromycin-resistance cassette containing the tetracycline repressor was excised from the pLEW90 vector with XhoI and StuI endonucleases. This cassette was then used to replace the neomycin-resistance cassette from the pLEW13_TbMrf1_3′/5′UTRs_neomycin vector, which was digested with XhoI and SwaI restriction enzymes. The resulting construct pLEW13_TbMrf1_3′/5′UTRs_hygromycin was linearized with NotI and the dKO cassette was transfected into the verified TbMrf1_sKO cell line. Selection with 25 μg/ml of hygromycin resulted in stably transfected cell lines termed TbMrf1_dKO. To verify that both *TbMrf1* alleles had been replaced, isolated gDNA served as a PCR template to produce amplicons with primer pairs that bridge the 5′ and 3′ integration sites of both selectable markers (5′UTR_ext_fw and dKO_rv; dKO_fw and 3′UTR_ext_rv) (Fig. 1a,b, Supplementary Table S1). The elimination of the *TbMrf1* open reading frame (ORF) from the genome was confirmed by PCR using ORF-specific primers (TbMrf1_fw and rv).

A conditional knockout (cKO) cell line with heterologous expression of a C-terminal 3xV5-tagged TbMrf1 in the dKO TbMrf1 7wk background was also generated. The TbMrf1 ORF was amplified from gDNA with the primers TbMrf1 cKO_fw and TbMrf1 cKO_rv (Supplementary Table S1). The resulting amplicon was digested with BamHI and HindIII before being ligated into a similarly digested pT7-3V5-PAC vector containing the puromycin resistance gene (PAC)^54^. The sequenced-verified construct was transfected into the dKO TbMrf1 7wk cell line and the transgenic trypanosomes were selected with 0.1 μg/ml of puromycin. These selected transgenic parasites were termed cKO TbMrf1 once it was verified by Western blot that tetracycline induced cells displayed the ectopic V5 TbMrf1 protein. This regulatable expression system is possible due to the previous TbMrf1 allelic replacements with the T7 polymerase and the tetracycline repressor.

In a similar manner, a dKO TbMrf1 + V5 TbPth4 (Tb927.6.2500) transgenic cell line was generated using the following forward (fw) and reverse (rv) primers: TbPth4V5_fw and TbPth4V5_rv (Supplementary Table S1).

To silence the expression of the *TbPth4* gene by double stranded RNA interference (RNAi), we employed a construct that inducibly expresses a stem loop RNA with T7 RNA polymerase. The original pRPhyg-iSL vector (courtesy of Sam Alsford) was adapted to our needs by replacing the hygromycin resistance gene with the ble gene that provides resistance to phleomycin. A 501 nt fragment of *TbPth4* that includes a portion of the 5′UTR and CDS (−55 nt to 446 nt) was amplified with a primer pair containing the restrictions sites SmaI and XhoI on the forward oligonucleotide, while the reverse contained BamHI and HindIII sites (TbPth4iSL_fw and rv). By digesting the same amplicon with either SmaI and BamHI or XhoI and HindIII, it could be sequentially ligated into the vector so that it was inserted twice in a head-to-head orientation, which would create a hairpin structure upon tetracycline-induced transcription. The transfection of the™ NotI-linearized pRPphleo_TbPth4-iSL construct into SM BF cells and the dKO TbMrf1 7wk cell line resulted in the RNAi TbPth4 and dKO TbMrf1 + RNAi TbPth4 cell lines, respectively. Both of these transgenic cell lines were actively selected using 2.5 μg/ml of phleomycin.

### Subcellular fractionation followed by carbonate extraction

Na_2_CO_3_ extraction of mt membranes was adapted from a previously published protocols^29,55^.

Briefly, mt vesicles from 5 × 10^8^ cells were isolated by hypotonic lysis. The mt pellet was further treated with digitonin (80 μg/ml) for 15 min on ice to disrupt the mt outer membrane. The material was then cleared by centrifugation (12,000 g, 20 min, 4 °C) and the pelleted mitoplasts were resuspended in 0.1 M Na_2_CO_3_ buffer (pH 11.5) and incubated for 30 min on ice. A final ultracentrifugation step (100,000 g, 4 °C for 1 hr, SW50Ti rotor) resulted in a supernatant comprised of proteins from the mt matrix, including stripped peripheral membrane proteins, and a pellet containing integral proteins isolated from the mt membrane fraction.

### Glycerol gradient fractionation

Whole cell lysates were prepared by resuspending 5 × 10^8^ BF cells in 300 µl of lysis buffer (30 mM HEPES pH 7.3, 12 mM MgCl_2_,120 mM KCl, 1% Nonidet NP40, 1 mM DTT) supplemented with 2 × EDTA-free protease inhibitors (Roche) and RNAseOUT RNAse inhibitor. Lysed cells were treated with TURBO DNAse for 15 minutes on ice and pelleted (21,000 g for 15 min at 4 °C). In the meantime, linear 10–30% glycerol gradients were prepared in thin-wall tubes (Beckman) using a Gradient Station (Biocomp) according to the manufacturer´s protocol. The cleared supernatant (200 μl) was loaded onto the glycerol gradient and centrifuged (38,000 g for 4 hours at 4 °C) in a SW 41 Ti rotor (Beckman Coulter). The Gradient Station was then used to collect 500 µl fractions that were used to isolate total RNA using a standard phenol-chloroform extraction protocol.

### Isolation of total RNA, reverse transcription, and qPCR

Quantitative reverse transcription PCR (RT-qPCR) was performed using a Light Cycler^®^ 480 system (Roche) according to the manufacturer’s recommendations. Briefly, total RNA from 1 × 10^7^ BF cells was extracted as described earlier and 10 μg were treated with TURBO DNAse (Ambion). The excess DNase was then removed using an RNAeasy kit (Qiagene). Following the manufacturer’s instructions, two micrograms of total RNA was reverse transcribed using the TaqMan Reverse Transcription kit (ABI). 100 ng of the resulting cDNA (2 μl) was mixed with SYBR Green (Applied Biosystems) and specific primers for either the TbPth4 transcript or the internal control transcripts that included 18S rDNA and β-tubulin (Supplementary Table S1). The samples were analyzed on a Light Cycler 480 (Roche) and the relative fold changes in the target amplicons were determined using the Pfaffl method, with the PCR efficiencies calculated by linear regression^56^.

### SDS-PAGE and Western blot analysis

Protein samples were separated by SDS-PAGE on a 12% Tri-Glycine acrylamide gel, blotted onto a PVDF membrane (BioRad) and probed with the appropriate monoclonal (mAb) or polyclonal (pAb) antibody. This was followed by incubation with a secondary HRP-conjugated anti-rabbit or anti-mouse antibody (1:2000, BioRad). Proteins were visualized using the Clarity ECL system (Bio-Rad) on a ChemiDoc instrument (BioRad). The Page Ruler pre-stained protein ladder (Thermo Scientific) was used to determine the size of detected bands. Primary antibodies used in this study included the mAb anti-V5 epitope tag (1:2000, Invitrogen), mAb anti-mtHsp70 (1:2000)^57^, pAb anti-MRP1 (1:1000)^58^, pAb anti-AAC (1:2000)^59^, and pAb anti-enolase (1:1000)^60^. Additionally, the following pAbs were produced in our laboratory: anti-APRT (1:500), anti-ATPaseTb2 (1:1000), anti-OSCP (1:500), anti-β subunit (1:2000) and anti-p18 (1:1000)^29^.

### Light blue native electrophoresis (BN PAGE)

Hypotonically isolated crude mt vesicles from 5 × 10^8^ BF trypanosomes were resuspended in 1 M aminocaproic acid (ACA) and lysed with 2% dodecyl-maltoside for one hour on ice. The samples were pelleted at 16,000 g for 30 min and the protein concentrations were determined by a Bradford assay. Equal amounts of protein for each sample were mixed with loading dye (0.5 M ACA, 5% w/v commassie G-250) and loaded onto a 3%–12% BisTris native gradient acrylamide gel. Two different native running buffers were used, a cathode buffer (50 mM tricine, 15 mM BisTris, 0.002% of Coommassie Blue) and an anode buffer (50 mM BisTris), both at pH 7. After electrophoresis (3 hr, 100 V, 4 °C), the resolved mt proteins were transferred onto a nitrocellulose membrane (90 min, 90 V, 4 °C, stirring) and probed with specific pAbs against F_o_F_1_-ATPase subunits: β, OSCP, ATPaseTb2, and p18.

### Mt membrane potential (Δψ_m_) measurement

The Δψ_m_ was determined utilizing the red-fluorescent stain tetramethylrhodamine ethyl ester (TMRE, Invitrogen). Cells in the exponential growth phase were stained with 60 nM of the dye for 30 min at 37 °C. Each time, 1 × 10^5^ cells were pelleted (1,300 g, 10 min, room temperature), resuspended in 1 ml of PBS (pH 7.4) and immediately analyzed by flow cytometry (BD FACS Canto II Instrument). For each sample, 10,000 fluorescent events were collected. Treatment with the protonophore FCCP (20 μM) for 10 min was used as a control for mt membrane depolarization. Data were evaluated using BD FACSDiva (BD Company) software.

### Drug sensitivity assay

Drug sensitivity assays using the resazurin sodium salt dye (Alamar Blue assay) were performed according to a published protocol^61^. Tested inhibitors dissolved in HMI-9 media (i.e. oligomycin and carboxyatractyloside, SIGMA) were serially diluted 2 fold across each column of a 96-well plate. Individiual wells were then seeded with either 5 × 10^3^ BF 427 cells or 1 × 10^4^ dKO TbMrf11wk/7wk cells, resulting in a total volume of 200 µl of HMI-9 media. The plates were incubated for 72 hours at 37 °C with 5% CO_2_. Finally, 20 µl of resazurin (0.125 mg/ml in 1xPBS, pH 7.4) was added to each well and the plate was further incubated for 8 hours. Fluorescence was measured on the Infinite M100 plate reader (Tecan) at excitation and emission wavelengths of 560 and 590 nm, respectively. Using GraphPad Prism 5.0, data were analyzed by non-linear regression with a variable slope.

### Survival analysis in an animal model

Three groups of 5 female BALB/c mice were infected via a 200 µl intraperitoneal injection of 1 × 10^5^ BF 427, dKO TbMrf11wk or 7wk trypanosomes. Parasitemia levels of individual animals were monitored daily for 7 days. Blood samples from a tail prick were diluted 100 times in TrypFix solution (1X SSC, 3.7% formaldehyde) and the number of parasites were quantified using an improved Neubauer haemocytometer. Mice displaying impaired health and/or a parasite load >10^8^ cells/ml of blood were euthanized. Survival of the experimental group was visually monitored for up to 30 days. The data were analyzed with the built-in survival analysis on GraphPad Prism 5.0.

All data generated or analyzed during this study are included in this published article (and its Supplementary Information file).

## Electronic supplementary material

Supplementary Information
