# Supplementary material for: Cultured bloodstream Trypanosoma brucei adapt to life without mitochondrial translation release factor 1
Source: Sci Rep. 2018 Mar 23;8:5135. doi: 10.1038/s41598-018-23472-6 (PMC5865105; doi:10.1038/s41598-018-23472-6)
Supplement: Supplementary file 1 — Supplementary Information [file 41598_2018_23472_MOESM1_ESM.pdf]

Supplementary Information

Cultured bloodstream *Trypanosoma brucei* adapt to life without mitochondrial translation release factor 1

Michaela Procházková, [a,b]* Brian Panicucci,[a] Alena Zíková [a,b]#

Institute of Parasitology, Biology Centre ASCR, Ceske Budejovice, Czech Republic[a]; Faculty of Science, University of South Bohemia, Ceske Budejovice, Czech Republic[b]

Running title: Bloodstream *T. brucei* mitochondrial translation

#Address correspondence to Alena Zíková, azikova@paru.cas.cz

Phone: (420)-38-777-5482; Fax: (420)-38-531-0388

*Present address: Central European Institute of Technology, Masaryk University, Brno, Czech Republic

Key words: Trypanosoma, mitochondrion, RNAi, mitochondrial translation, mitoribosome, FoF1-ATPase

Supplementary Figure S1. Lack of TbMrf1 did not result in cells without kinetoplast DNA.

(a) Left panels: fluorescence microscopy of DAPI-stained BF 427, dKO TbMrf1 1wk and 7wk cells. Right panels: the DNA content and single tubular mitochondrion was visualized with DAPI and with a fluorescein isothiocyanate (FITC)-conjugated secondary antibody that recognizes a polyclonal primary antibody detecting F_1-ATPase subunit β, respectively. DIC - differential interference contrast; n – nucluei; k – kinetoplast.

(b) Quantification of the microscopy images based on the number of nuclei and kinetoplasts in more than 200 cells per cell line. Only normal cell types either in G1/S (1N1K) and G2/M (1N2K) phases or undergoing cytokinesis (2N2K) were detected.

Supplementary Figure S2. Ectopic overexpression of TbMrf1 in the background of TbMrf1 dKO decreases the sensitivity to oligomycin and carboxyatractyloside inhibitors

(a) Whole cell lysates from BF 427 cultures and cKO TbMrf1 7wk cells either noninduced (-tet) or induced (+tet) with tetracycline for 48 hours were probed with a specific anti-V5 antibody. An anti-APRT antibody was used as a loading control.

(b) The oligomycin and carboxyatractyloside sensitivity of cKO TbMrf1 7wk +tet cells was determined by an Alamar Blue assay. The dose-response curves were calculated using GraphPad Prism. Error bars represent the standard deviation calculated from three independent experimental replicates.

Supplementary Figure S1

a

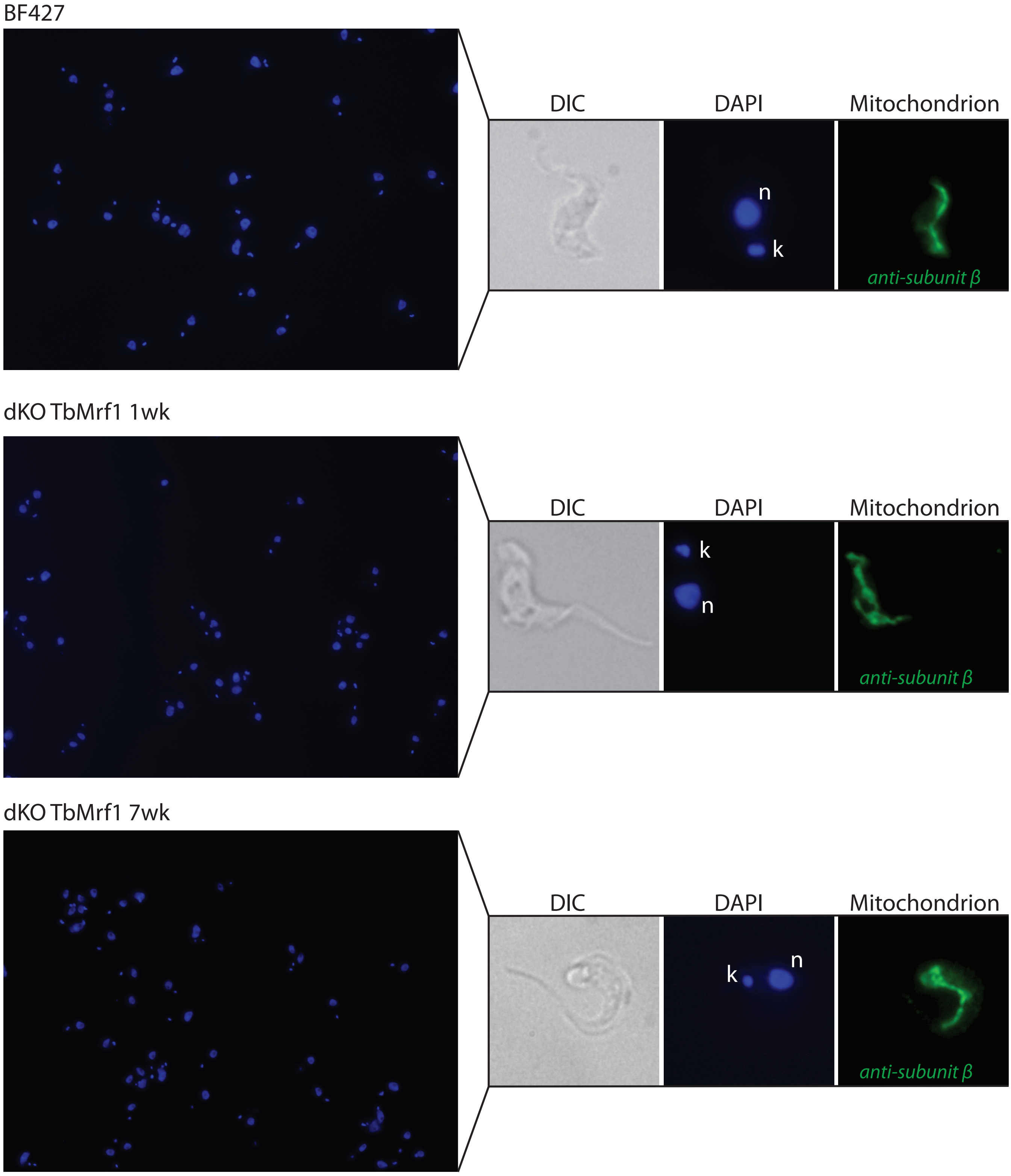

b

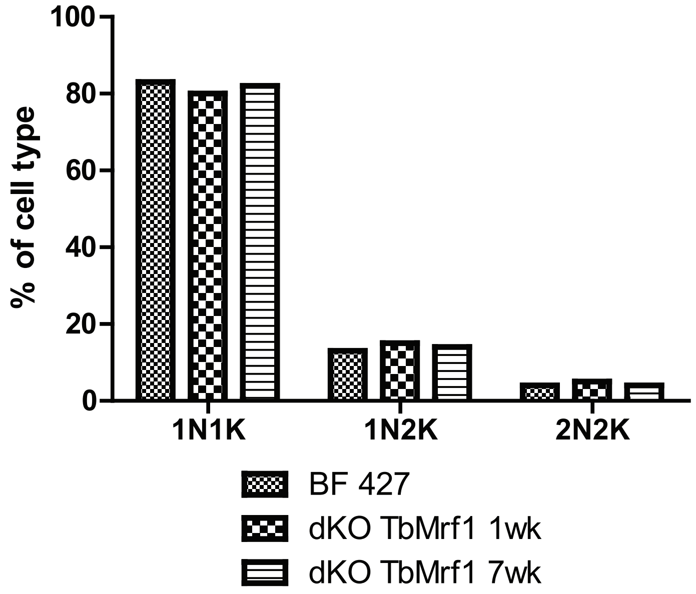

Supplementary Figure S2

a

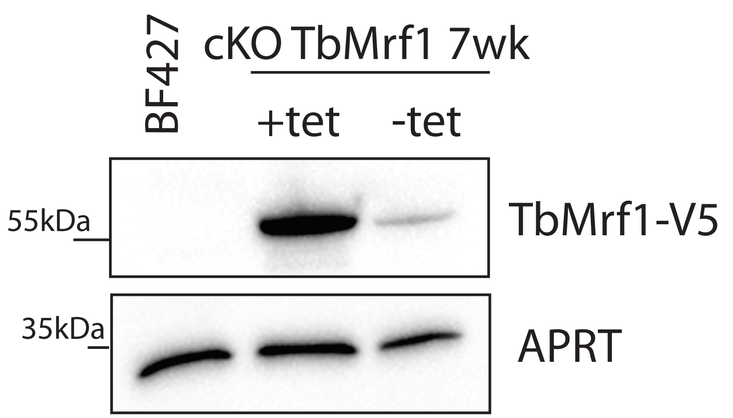

b

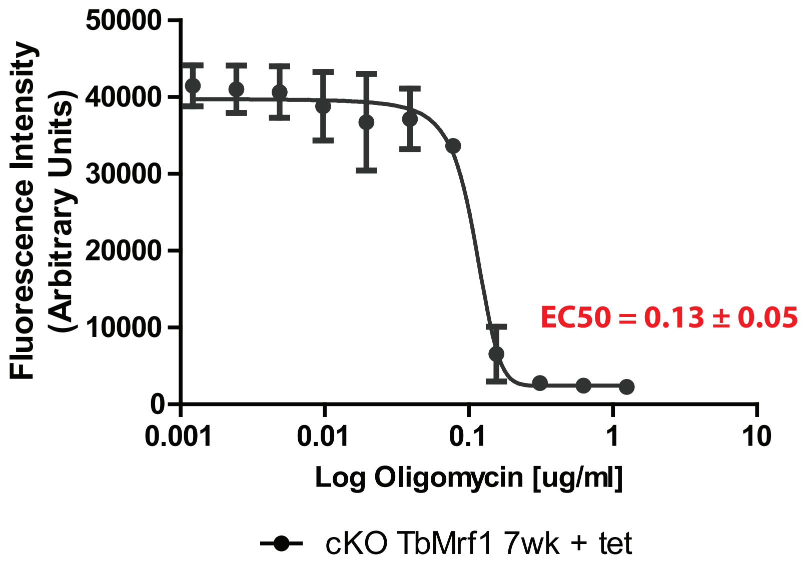

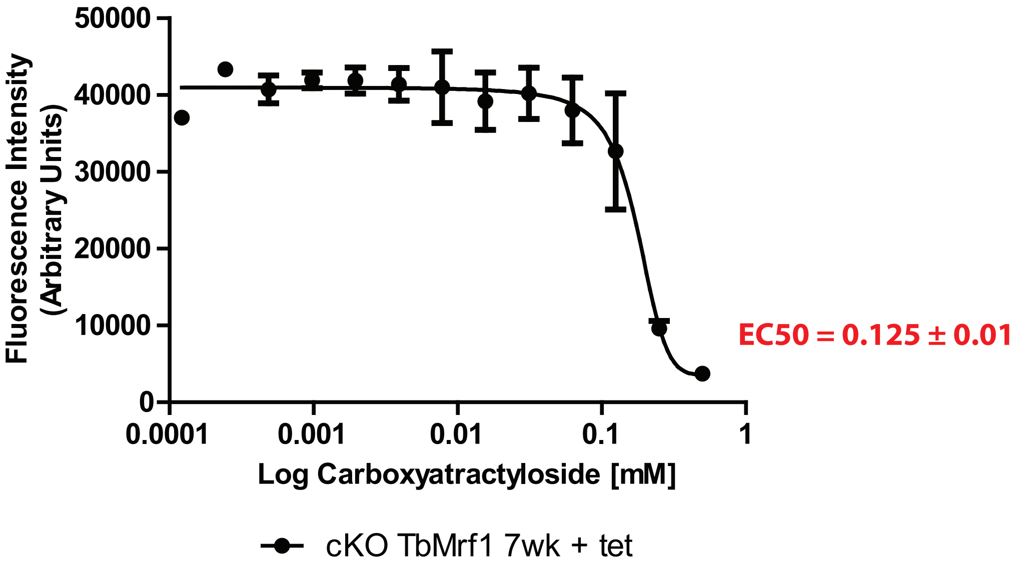

Supplementary Table S1. List of oligonucleotides

Primer name	Primer sequence
**5'UTR_fw**	TATGCGGCCGCTGTTCTATAACCGAGGACG
**5'UTR_rv**	AGGACGCGTCTCGAGATTCTTGGAATTGCTTGTACG
**3'UTR_fw**	GGGTCTAGAATTTAAATGAGAAGACGTGGGTGCCTAG
**3'UTR_rv**	TATAGGCCTGCGGCCGCTTGCCACTTTGGATCTGGG
**5'UTR_ext_fw**	AGTATTGGGCTATCGTCAGG
**3'UTR_ext_rv**	GAGGCAAATATGGTGAGAGC
**sKO_fw**	CTTGCCGAATATCATGGTGG
**sKO_rv**	GTAAATCCGGATCAGATCAGC
**dKO_fw**	TACTCGCCGATAGTGGAAACC
**dKO_rv**	CGCGATGACTTAGTAAAGCAC
**TbMrf1_fw**	ATAGGATCCATGAGGAATGCCCCATTGCTC
**TbMrf1_rv**	GCGAAGCTTTTACGTAACACAGTTGAAGTCG
**TbMrf1 cKO_fw**	CACAAGCTTATGAGGAATGCCC
**TbMrf1 cKO_rv**	CACGGATCCCGTAACACAGTTGAAG
**β-tubulinqPCR_fw**	TTCCGCACCCTGAAACTGA
**β-tubulinqPCR_rv**	TGACGCCGGACACAACAG
**18S rRNA_fw**	CGGAATGGCACCACAAGAC
**18S rRNA_rv**	TGGTAAAGTTCCCCGTGTTGA
**12S rRNA_fw**	GGGCAAGTCCTACTCTCCTTTACAAAG
**12S rRNA_rv**	TGAACAATCAATCATGGTAATAAGTAGACGATG
**9S rRNA_fw**	ATTAGATTGTTTTGTTAATGCTATTAGATG
**9S rRNA_rv**	ACGGCTGGCATCCATTTC
**TbPth4qPCR_rv**	TCTTTCAGAAACGATATGTG
**TbPth4qPCR_fw**	CTTCAGCAGATGATTCAC
**TbPth4V5_fw**	GGGAAGCTTATGAGTTGGTTAACTCCTTCAGG
**TbPth4V5_rv**	AAAGGATCCCCACAGCCCTTTGCGAG
**TbPth4iSL_fw**	ATTCTCGAGCCCGGGCAGAGACGAGGTAATTTCC
**TbPth4iSL_rv**	ACCAAGCTTGGATCCTGATCAGCTGTGTCTCATCC
**TbMrf1qPCR_fw**	ATGCAAAGCAGTTCCAACGC
**TbMrf1qPCR_rv**	ACACGACGTACCAACAGTTG
